# Earth Closets

**Published:** 1869-08-15

**Authors:** 


### *Earth Closets.*

We have been favored by Sanitary Superintendent Ranch with an examination of a model of this new and exceedingly important invention, which he desires to introduce into general use in this city. In no city in the country, except, perhaps, New Orleans, does the subject of the disposition of the excrementitious matter demand more attention than in Chicago; having been rejected by the system as worthless and hurtful. The primary object to be kept in view, in the consideration of the subject, would seem to be to prevent their return into the animal economy from which they had been ejected. To accomplish this object it is necessary to protect against contamination the air which we breathe and the water which we drink, both of which are continually contaminated, more or less directly, by every mode yet devised for the disposition of human excreta. A secondary, though still vitally important object to be considered is the useful or profitable disposition of these matters, that they may become, instead of a nuisance, a source of wealth, by returning food to the earth that we may draw therefrom increased supplies of food for man.

If this invention will perform what it promises, we believe it will greatly facilltate, if it does not secure the accomplishment of all these objects. It is certainly worth examination by every physician and householder.

